# Love Your Country: EEG Evidence of Actor Preferences of Audiences in Patriotic Movies

**DOI:** 10.3389/fpsyg.2021.717025

**Published:** 2021-07-16

**Authors:** Lian Zhu, Yufei Wu

**Affiliations:** School of Journalism and Communication, Shanghai International Studies University, Shanghai, China

**Keywords:** audience emotions, actor preferences, cognitive conflict, patriotic movies, EEG

## Abstract

Movie watching is one of the common ways to spark love for the country. A good patriotic movie can arouse love and pride, encourage people to stand by their countries, and reinforce a sense of national belonging. To evoke audience emotion and enhance patriotism, the choice of actors is fundamental and is a dilemma for film producers. In this exploratory study, an electroencephalogram (EEG) with a rating task was used to investigate how actor types (i.e., skilled vs. publicity) in patriotic movies modulate the willingness of audiences to watch a film and their emotional responses. Behavioral results showed that audiences are more willing to watch patriotic movies starring skilled actors than to watch patriotic movies starring publicity actors. Furthermore, brain results indicated that smaller P3 and late positive potential (LPP) were elicited in response to skilled actors than to publicity actors in patriotic movies. A larger theta oscillation was also observed with skilled actors than with publicity actors. These findings demonstrate that the willingness of audiences to watch a movie is deeply affected by actor types in patriotic films. Specifically, skilled actors engage audiences emotionally, more so than publicity actors, and increase the popularity of patriotic movies. This study is the first to employ neuroscientific technology to study movie casting, which advances film studies with careful scientific measurements and a possible new direction.

La première des vertus est le dévouement à la patrie.

*Napoléon Bonaparte*

## Introduction

Patriotism is an important social concern. It is passion and pride for the motherland, and respect for our roots, which holds the whole nation together. We will have tears in our eyes seeing soldiers put their lives in danger to fight for the motherland. We will also feel anger seeing the country led down the wrong path by the wrong people. Movie watching is one of the common ways to spark this love for the country in our daily lives. When emotions of an audience are well-engaged, a good patriotic movie can arouse love and pride, encourage people to stand by their countries, and reinforce a sense of national belonging. However, not all patriotic movies manage to evoke a strong audience reaction and perform well at the box office.

The cast of the movie may be an important factor that caused this difference. It has been acknowledged that one of the most important means to engage audiences emotionally, and in turn, increase their willingness to watch a film, is to hire suitable stars (Vincendeau, 2000; Marich, 2009; Gunter, 2018). For example, Austin discussed the star/character interface of different actors who have played Batman and argued that actors with a well-built appearance asserted Batman “authenticity,” while those who had a non-matched persona would lead to well-publicized complaints from fans and commentators (Austin, 2003). Furthermore, Hofmann suggested that actors can be assigned to one of two generic star profiles: skilled and publicity. Skilled actors are famous for their acting ability, whereas publicity actors are often chosen for their physical appearance (Hofmann and Opitz, 2019). Previous studies have explained different functions of drawing power of stars (Chisholm, 2004; Joshi, 2015; Suárez-Vázquez, 2015; Hennig-Thurau and Houston, 2019). When it comes to understanding the willingness of an audience to watch a patriotic movie, it is interesting to ask what is more important—Is it the professional skills or the outer appearance of actors?

For skilled stars, it is acknowledged that they can signal production quality. Skilled stars are famous for their outstanding acting abilities and are often capable of winning prestigious awards. They improve the artistic value of the patriotic movies in which they appear, and they shape more subtle and demanding character roles to arouse audience emotions (Hofmann, 2019). Since artistic quality is what patriotic movies have at stake (Yang, 2017), skilled stars may increase the willingness of an audience to watch a patriotic movie by making this type of movie more artistically engaging and creating the kind of cinematographic experience that film connoisseurs look for. They may also be good with the realistic storyline that patriotic movies often deploy, catering to the cognitive needs of the audience (Hofmann, 2019), and have a positive influence on the market performance of movie (Desai and Basuroy, 2005). Furthermore, it has been argued that, since patriotic movies are a less familiar genre, they require strong, skilled actors to bring subtle emotional touches, boost the overall aesthetic quality of the production (Yang, 2017), and, with it, the willingness of the audience to watch, more so than in the more popular and familiar genre movies (Desai and Basuroy, 2005).

On the other hand, publicity stars are more suited to create a media buzz and make the movies more visible for a mass audience (Hofmann, 2019). They add dynamism to market campaigns and provide opportunities for gossip and small talk, but their entertainment function does not seem to be compatible with patriotic movies. This is because publicity stars are famous mainly for their physical appearance (Hofmann and Opitz, 2019), which may not be an important element for patriotic movies. Moreover, publicity stars are more likely to have a relatively fixed screen persona, which may be easily associated with particular types of movies, such as comedy or romance, as well as the gossip they produce. We believed that this “mismatch” may engender a cognitive conflict for the audience. It may also distract people from the storyline and the emotional power of patriotic movies and, in turn, reduce willingness of the audience to watch. It is also suggested by previous studies that publicity stars are more suitable for movie genres that strive to offer an accessible and easily comprehensible entertainment experience for a mainstream audience (Giles, 2000; McCutcheon et al., 2003; Cheung and Yue, 2011). To sum up, we hypothesize that audiences prefer skilled actors to publicity actors in patriotic movies.

Beyond behavioral tests, neural techniques are well-placed to measure emotional arousal and cognitive conflict. Film studies have embraced neural techniques in recent years in order to better understand the emotional reactions of audiences. Electroencephalography (EEG) offers high temporal resolution (Xu et al., 2018), which makes it a valuable technique to distinguish early perceptual reactivity from more complex and elaborated emotional processes (Gardener et al., 2013). A recent EEG study, for example, found a relatively high proportion of relaxed alpha waves when people watched television and video content, confirming that viewing induces a state of pleasant and wakeful relaxation (Barwise et al., 2019). In the present study, we also use EEG to measure the emotional responses of audiences to two types of actors in patriotic movies, with an attempt to examine the underlying neural basis of the effects of actor types on their willingness to watch a patriotic movie. To the best of our knowledge, this study is the first to use neural techniques to differentiate the influences of actors on the willingness of an audience to watch a movie.

Previous studies have shown that the EEG and some late event-related potential (ERP) components, such as the theta ERS (Mitchell et al., 2008), P3 (Hajcak et al., 2010), and late positive potential (LPP) (Cuthbert et al., 2000), are sophisticated ways to process emotional visual stimuli and detect the emotional effect. In this study, we examine the time-frequency power and two ERP components: the theta ERS, P3, and LPP. The P3 is a positive-going waveform over the centro-parietal sites, which peaks around 250–450 ms after the stimuli onset. It was reported that affective and motivational stimuli elicit enhanced P3 amplitudes (Palomba et al., 1997; Cuthbert et al., 2000; Di Russo et al., 2006; Hajcak et al., 2010). In addition, some empirical evidence suggested that long-lasting, positive LPP, which reaches its maximum around 500–700 ms (Olofsson et al., 2008; Hajcak et al., 2010), is a more reliable, replicable, and temporally sensitive indicator of emotional processing (Cuthbert et al., 2000; Schupp et al., 2003, 2007; Hajcak et al., 2006). According to previous studies, in the context of affective perceptual processing, the LPP amplitude is reported to reflect sustained and enhanced attention allocation and motivational significance to emotional visual stimuli (Bradley et al., 2003; Lang and Bradley, 2009). Specifically, the higher amplitude of the P3 and LPP tends to occur more often for emotionally significant stimuli (pleasant and unpleasant) than for neutral visual stimuli (Carretié et al., 1997; Olofsson et al., 2008). In other words, the P3 and LPP emotional effect may be dependent on how a person appraises an emotional stimulus and is likely to reflect the motivations underlying behaviors (Conroy and Polich, 2007; Zhang et al., 2019). Thus, in this study, according to the behavioral hypothesis, we predict that, in the patriotic movie condition, the not well-matched publicity stars would gain a greater allocation of attention for the emotion arousal effect than the fit skilled actors, indicating the top-down mechanism that is involved in emotion regulation (McRae et al., 2012), which is reflected in larger LPP and P3 amplitude.

In addition to the EEG and ERPs, we also investigated whether the event-related desynchronization or synchronization (ERD or ERS) can reflect the evaluation of actors and films. Theta band oscillation (4–8 Hz) is a frequently examined functional signaling in the neural system, which is related to emotion and motivation (Başar et al., 2006). It was reported that motivational and affective stimuli (e.g., erotic and threatening pictures) induce larger theta ERS regions when compared with neutral stimuli, indicating that theta oscillation mediates motivated attention (Aftanas et al., 2008). Furthermore, larger theta ERS was observed to be positively correlated with pleasant emotional experience and negatively correlated with negative emotions (Aftanas and Golocheikine, 2001; Sammler et al., 2007). A previous study also confirmed that the presentation of positive affective movies yields an increment in theta power, while a power decrease was observed in response to emotionally negative movies (Aftanas et al., 1998). In line with the hypothesis proposed above, we predict that, in the patriotic movie condition, larger theta ERS would be observed for the skilled actors than for the publicity actors.

Overall, we aimed to use EEG techniques to explore how actor types in patriotic movies modulate film-watching willingness and emotional responses of an audience. We supposed that audiences would prefer skilled actors to publicity actors in patriotic movies, reflecting in behavioral rating scores and deflection of P3 and LPP amplitude, and theta ERS.

## Materials and Methods

### Participants

Fifty female (mean age = 21.9, *SD* = 2.1) students from Shanghai International Studies University participated in this study, and four of them were discarded due to inadequate artifact-free ERP trials. All participants are right-handed with a normal or a corrected-to-normal visual acuity and reported no history of neurological or psychiatric disorders or head trauma. None of them were fans of our stimuli actors. Before the research protocol began, all participants received experimental instructions and gave written informed consent. The experiment was conducted under the Declaration of Helsinki (World Medical Association, 2013) and was approved by the Ethics Committee. Each participant was compensated with 50 RMB (about US $7.50) for the study when finished.

### Materials and Procedure

A 2 (movie type) × 2 (actor type) study was conducted. Forty movies (patriotic vs. non-patriotic) and 20 actors (skilled vs. publicity) were paired up. A total of 800 pairs constituted the stimuli pool, and 160 stimuli (40 per condition) randomly from the stimuli pool were shown in every experiment with a programmatically fixed occurrence number of each movie title (four times) and actor name (eight times).

In the present study, participants were asked to identify the movie type from their titles. A movie title is one of the first pieces of information delivered to potential viewers, and it can provide information for moviegoers to interpret the genre or storyline of the movie (Bae and Kim, 2019). Eighty fictional titles were brainstormed (i.e., 40 patriotic and 40 non-patriotic), and an independent cohort of 40 students was recruited. All titles were rated by the degree of patriotism on a 7-point Likert scale (1 = not at all, 7 = very much). Consequently, the top 20 rated titles (mean score = 6.17, *SD* = 0.31) were categorized as patriotic, and the lowest scored 20 titles (mean score = 2.98, *SD* = 0.65) were the non-patriotic [*t*-test: *t*_(19)_ = 40.146, *p* = 0.000]. The length of all film titles used was within three to five Chinese characters.

As for actor selection, 48 Chinese actors (24 per type) whose ages ranged from 26 to 39 were chosen. Fifty-eight students (15 males) were recruited to rate them based on their acting skills, publicity, and familiarity on a 5-point Likert scale. After that, actors were selected according to three criteria. First, the scores they gained should have a small SD. Second, they should gain high scores (above average) in one actor type and low scores (below average) in another. Third, they should have medium familiarity (within plus or minus one SD on average) to assure a similar familiarity level. As a result, 20 actors (mean age = 32.6, *SD* = 4.41; mean familiarity = 3.37, *SD* = 0.28), namely, 10 skilled actors (mean skilled score = 3.91, *SD* = 0.19; mean publicity score = 2.72, *SD* = 0.38) and 10 publicity actors (mean skilled score = 2.79, SD = 0.27; mean publicity score = 3.77, *SD* = 0.25), were selected [skilled *t*-test: *t*_(9)_ = 7.063, *p* = 0.000; publicity *t*-test: *t*_(9)_ = −8.639, *p* = 0.000]. No significant familiarity difference was found across the two types [*t*_(9)_ = 0.580, *p* = 0.576].

After seating the participants in an armchair ~70 cm in front of the computer screen, a rating task, programmed and executed with E-prime 3.0 (Science Plus Group, Groningen, The Netherlands), was performed. Five practice trials (not considered for the analysis) were conducted to acquaint participants with the procedure before starting.

**Figure 1:**
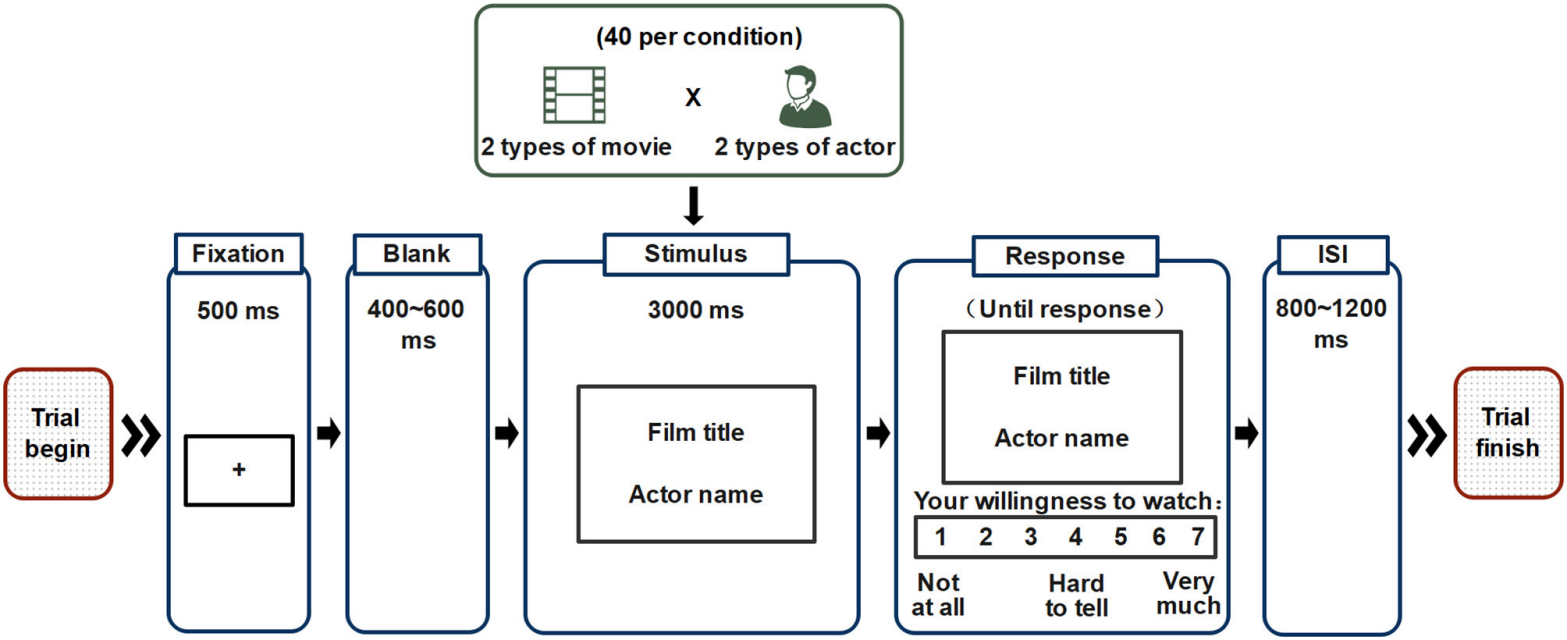
Example of trial sequence. The movie title and actor name were shown to subjects after a fixation point and a blank screen in each trial. Then, subjects were asked to perform a rating task.

As shown in Figure 1, each trial began with a fixation point (duration = 500 ms) followed by a blank screen (duration = 400–600 ms). A movie title and the name of an actor were then displayed simultaneously at the center of the screen (duration = 3,000 ms). Next, a 7-point rating bar (1 = not at all, 7 = very much) appeared below, asking participants to rate their willingness to watch this movie starring this actor by pressing the numeric keyboard. After the response, the interstimulus interval (ISI), a blank screen was presented randomly from 800 to 1,200 ms as a link between two trials.

When finished, participants were invited to fill out a self-reported questionnaire to record their demographic information and general movie consumption habits. They were also asked to rate their interest in different attributes of movies and familiarity with patriotic films.

### Recording of EEG

The EEG was recorded (band-pass = 0.1–100 Hz, sampling rate = 500 Hz) using a 32-channel EEG system (Brain Products GmbH, Gilching, Germany). Distributed according to the 10–20 international system (Sharbrough et al., 1991), all Ag/AgCl electrodes were mounted in the caps, and their impedances were maintained below 10 kΩ. Electrode FCz was used for online EEG reference. The correct functioning of all electrodes was verified before starting the recording.

### Data Analysis of EEG

The data were processed using the EEGLAB (Delorme and Makeig, 2004) open-source software version 14.1.1, running on MATLAB R2016a (The Mathworks Inc.). The EEG signal was algebraically re-referenced offline to the average of the left and right mastoids. A basic FIR filter between 0.1 and 30 Hz was then applied. Continuous EEG was segmented into ERP epochs, which were time-locked from 200 ms before the stimuli screen to 800 ms after the onset. The baseline for ERP measurements was the mean voltage over 200 ms before the stimuli onset. For correcting artifacts caused by eyeblinks, eye movements, or muscle potentials, the independent component analysis (ICA) was applied with the ADJUST plugin (Mognon et al., 2011) for EEGLAB as an accessory tool. After that, trials contaminated by remaining artifacts that exceeded ± 100 μV at any electrode were excluded from averaging. ERP was then averaged under each condition. Participants were removed from the following analysis if any of the condition averages were <30 trials due to excessive artifacts. Ultimately, 46 out of 50 participants entered the subsequent studies.

Based on the P3 (Johnson, 1988; Polich, 2007; Zhang et al., 2017) and LPP (Schupp et al., 2000; Liu et al., 2012; Hajcak and Foti, 2020) results in previous studies, as well as visual inspection of the waveforms and their topographical distributions, we carried out an analysis of the P3 using the mean amplitude over 300–390 ms in the parietal area (P3, Pz, P4), and the 550–700 ms time window from five electrodes (P3, Pz, P4, CP1, and CP2) was chosen for LPP analysis. The repeated-measures ANOVA was used to examine the effects of movie types (patriotic vs. non-patriotic) and actor types (skilled vs. publicity) on component amplitudes.

As for the time-frequency analysis, the artifact-free EEG data within each condition were decomposed into time-frequency representations with a frequency range from 1 to 30 Hz in 50 logarithmically spaced steps in which the power spectrum of the EEG signal was multiplied by the power spectrum of complex Morlet wavelets (*e*^*j*2π*tf*^ *e*^−*t*^2^/2σ^2^^). In this formula, *t* represents the time, *f* represents the frequency, and σ is the width of each frequency band, set as 3–7 logarithmically spaced cycles to allow a trade-off of temporal and frequency resolution. After that, we applied the inverse FFT (fast Fourier transform). The modulus of the resulting complex signal Z(real [z(t)]^2^ + imag [z(t)]^2^) was defined as power. The TFRs of each condition of each participant were averaged and transformed using a decibel (dB) scale. For normalization, the activity from −300 to −100 ms before the stimuli was used as the baseline for each frequency with the equation: *dB power* = 10 × log 10(*power*/*baseline*).

Since the frontal midline theta (FM-theta) activity was recognized as a crucial indicator of emotional state (Aftanas and Golocheikine, 2001; Kubota et al., 2001), the electrode Fz, where the FM-theta is generally maximal (Yamamoto and Matsuoka, 1990; Mitchell et al., 2008), was chosen for analysis. Additionally, a complementary line of research has observed target-related theta ERS in the period 200–500 ms (Kolev et al., 1997; Yordanova and Kolev, 1998; Wang and Ding, 2011). Therefore, the mean magnitude of FM-theta-band activity (Fz) (frequency range = 4.1–5.8 Hz, time range = 330–380 ms) was delivered to a repeated-measures ANOVA with factors “movie type” and “actor type.” A simple-effect analysis would be performed once any significant interaction occurred. Greenhouse–Geisser correction (Greenhouse and Geisser, 1959) for repeated measures was applied for statistical analysis. The significance level was set as *p* < 0.05.

## Results

### Behavioral Results

**Figure 2:**
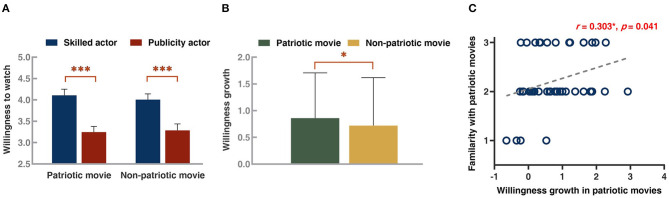
Behavioral results. **(A)** Average willingness under four conditions. **(B)** Willingness differences (skilled–publicity) in two movie types. **(C)** Scatter plots of Pearson's correlation coefficient analysis results between the familiarity level with patriotic movies and the willingness growth in such movies. The willingness growth represented the willingness difference (skilled–publicity) in each movie type. Blue, red, green, and yellow in bar graphs stood for skilled, publicity, patriotic, and non-patriotic. Error bar: standard error, *p < 0.05, ***p < 0.001.

A 2 (movie type: patriotic vs. non-patriotic) × 2 (actor type: skilled vs. publicity) repeated-measures ANOVA was conducted on the average willingness to watch (Figure 2A). This analysis revealed a significant main effect of actor type [mean ± standard error, skilled: 4.06 ± 0.13 vs. publicity: 3.27 ± 0.13, *F*_(1, 45)_ = 39.83, *p* = 0.000] with a more vital willingness in the skilled actor condition, but there was no significant main effect of movie type [*F*_(1, 45)_ = 0.08, *p* = 0.773]. Moreover, a significant interaction [*F*_(1, 45)_ = 5.68, *p* = 0.021] between movie type and actor type was found. When movie types were fixed, the simple-effects analysis confirmed a stronger willingness for skilled than for publicity actors in both patriotic movies [skilled: 4.11 ± 0.98 vs. publicity: 3.24 ± 0.91, *F*_(1, 45)_ = 47.565, *p* = 0.000] and non-patriotic movies [skilled: 4.01 ± 0.91 vs. publicity: 3.29 ± 1.04, *F*_(1, 45)_ = 29.617, *p* = 0.000].

Further analysis was conducted, in which we defined the average willingness difference (skilled–publicity) in each movie type as the willingness growth caused by skilled actors. The result illustrated a more significant willingness increase in patriotic movies than in non-patriotic movies [patriotic: 0.86 ± 0.12 vs. non-patriotic: 0.72 ± 0.13, *t*_(45)_ = 2.384, *p* = 0.021, Figure 2B]. Besides, we were curious about whether the watching experience might be related to actor preference in movie-watching. Pearson's correlation coefficient analysis was performed between the familiarity level with patriotic movies and the willingness growth in this type. A significant positive correlation was observed (*r* = 0.30, *p* = 0.041, Figure 2C), which meant the more familiar the participant was with patriotic movies, the larger was the growth of her willingness.

### Results of ERP: P3

**Figure 3:**
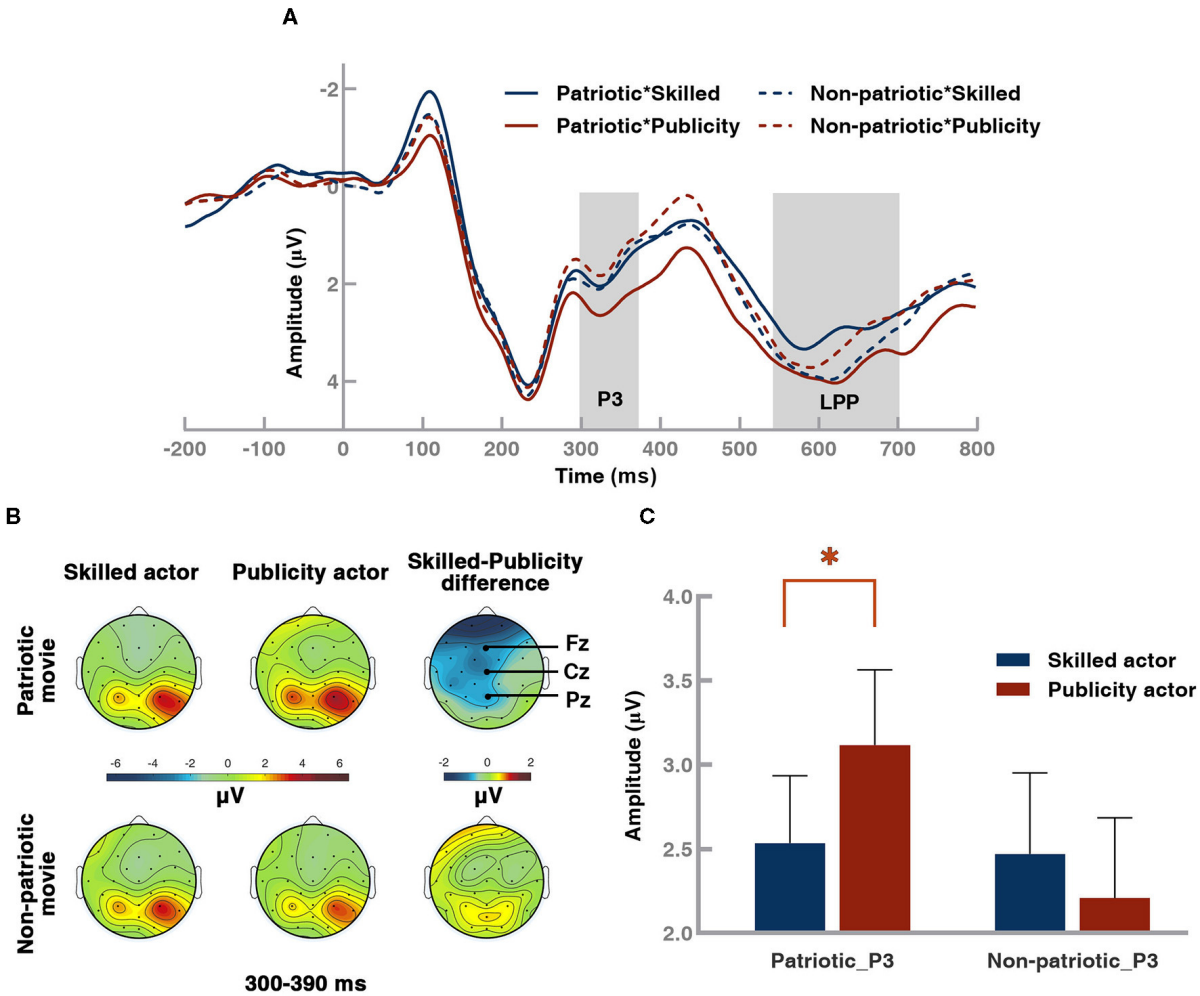
Event-related potential (ERP) waveforms from Pz, topography of grand-averaged P3 waveforms from Pz, and P3 results. **(A)** Grand-averaged ERP under four conditions: 2 movie types (patriotic: solid line, non-patriotic: dotted line) × 2 actor kinds (skilled: blue, publicity: red). **(B)** Topography of P3. **(C)** P3 averaged amplitudes of patriotic movies and non-patriotic movies starring different types of actors from the parietal scalp region. Blue represents the skilled actors, and red represents the publicity actors. The topography was the averaged potential of the 330–390 ms time window. Error bar: standard error, *p < 0.05.

Consistent with previous studies (Johnson, 1988; Polich, 2007; Zhang et al., 2017), a robust P3 component was observed in the parietal scalp region (Figures 3A,B). We analyzed the P3 using the mean amplitude over 300–390 ms from three electrodes (P3, Pz, and P4). A three-way 2 (movie type: patriotic vs. non-patriotic) × 2 (actor type: skilled vs. publicity) × 3 (electrode: P3/Pz /P4) ANOVA was conducted (bar plot of P3 amplitudes, see Figure 3C). There was a significant main effect of movie type [patriotic: 2.83 ± 0.40 vs. non-patriotic: 2.34 ± 0.45, *F*_(1, 45)_ = 4.611, *p* = 0.037] with a larger P3 amplitude induced by patriotic movies than by non-patriotic ones. A significant interaction effect between movie type and actor type [*F*_(1, 45)_ = 4.920, *p* = 0.032] was found, whereas no significant main effect of actor type [*F*_(1, 45)_ =0.388, *p* = 0.536] was observed. Further results of simple-effect analysis illustrated that the effect of actor type was significant [*F*_(1, 45)_ = 4.211, *p* = 0.046] when the movie type was fixed to patriotic movies. The mean amplitude of the P3 in response to publicity actors (3.12 ± 0.45 μV) was larger than that in response to skilled actors (2.54 ± 0.40 μV). However, the effect of actor type was not significant [*F*_(1, 45)_ = 0.552, *p* = 0.461] with non-patriotic movies.

### Results of ERP: LPP

**Figure 4:**
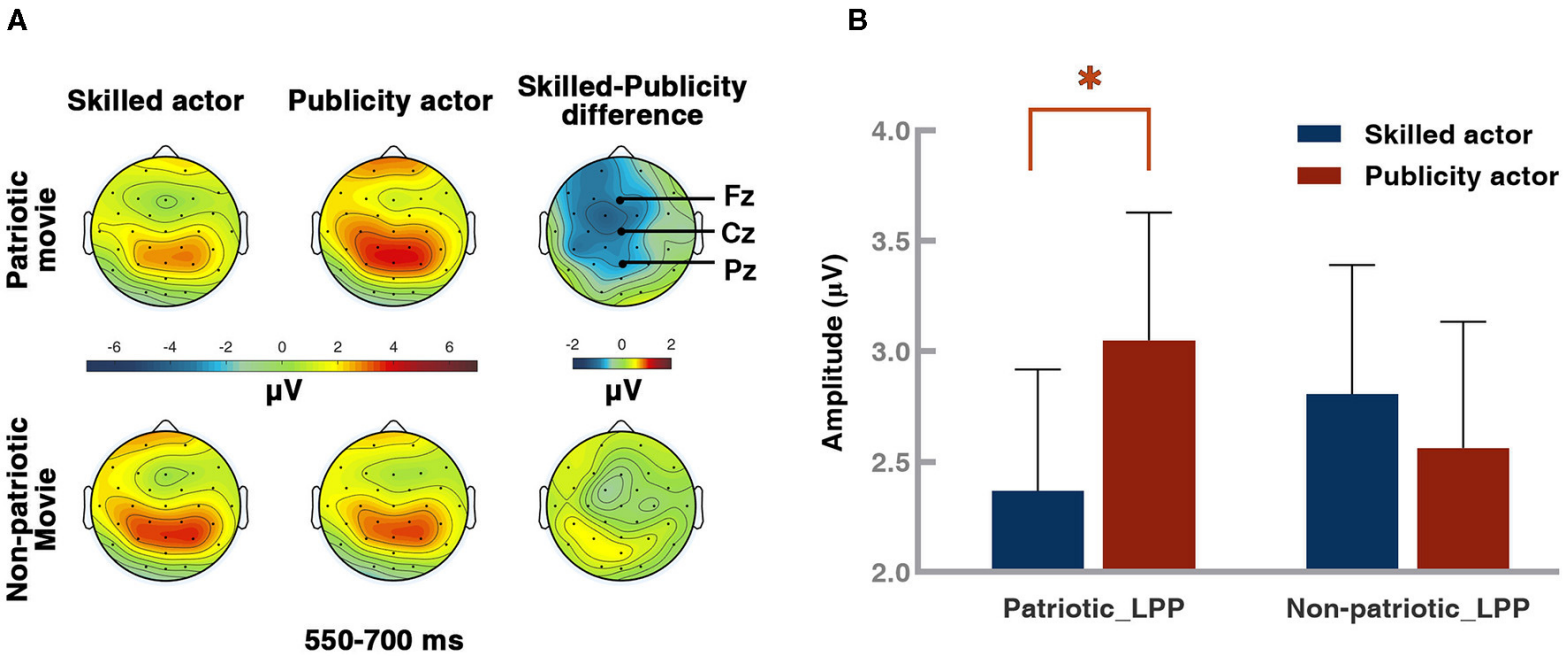
Topography of grand-averaged late positive potential (LPP) waveforms from Pz and LPP results. **(A)** Topography of LPP. **(B)** LPP-averaged amplitudes of patriotic movies and non-patriotic movies starring different types of actors from selected electrodes. The topography was the averaged potential of the 550–700 ms time window. Blue represents the skilled actors, and red represents the publicity actors. Error bar: standard error, *p < 0.05.

Based on the LPP results in previous studies (Schupp et al., 2000; Liu et al., 2012; Hajcak and Foti, 2020), as well as visual inspection of the waveforms and their topographical distribution (Figures 3A, 4A), the time window of 550–700 ms from five centro-parietal electrodes (P3, Pz, P4, CP1, and CP2) was chosen for LPP analysis. The results of a 2 (movie type: patriotic vs. non-patriotic) × 2 (actor type: skilled vs. publicity) × 5 (electrode: CP1/CP2/P3/Pz /P4) ANOVA, as shown in Figure 4B, revealed a significant interaction between movie type and actor type [*F*_(1, 45)_ = 6.416, *p* = 0.015], but no significant main effects of either movie type [*F*_(1, 45)_ = 0.010, *p* = 0.919] or actor type [*F*_(1, 45)_ 0.798 1, *p* = 0.376]. When the simple-effect analysis was performed, a significant effect of actor type was found in patriotic movies [*F*_(1, 45)_ = 5.341, *p* = 0.025]. More specifically, the amplitude of LPP elicited by the publicity actors (3.05 ± 0.58 μV) was significantly larger than that by the skilled actors (2.37 ± 0.55 μV). No significant difference was observed under the non-patriotic movie condition [*F*_(1, 45)_ = 0.597, *p* = 0.445].

**Figure 5:**
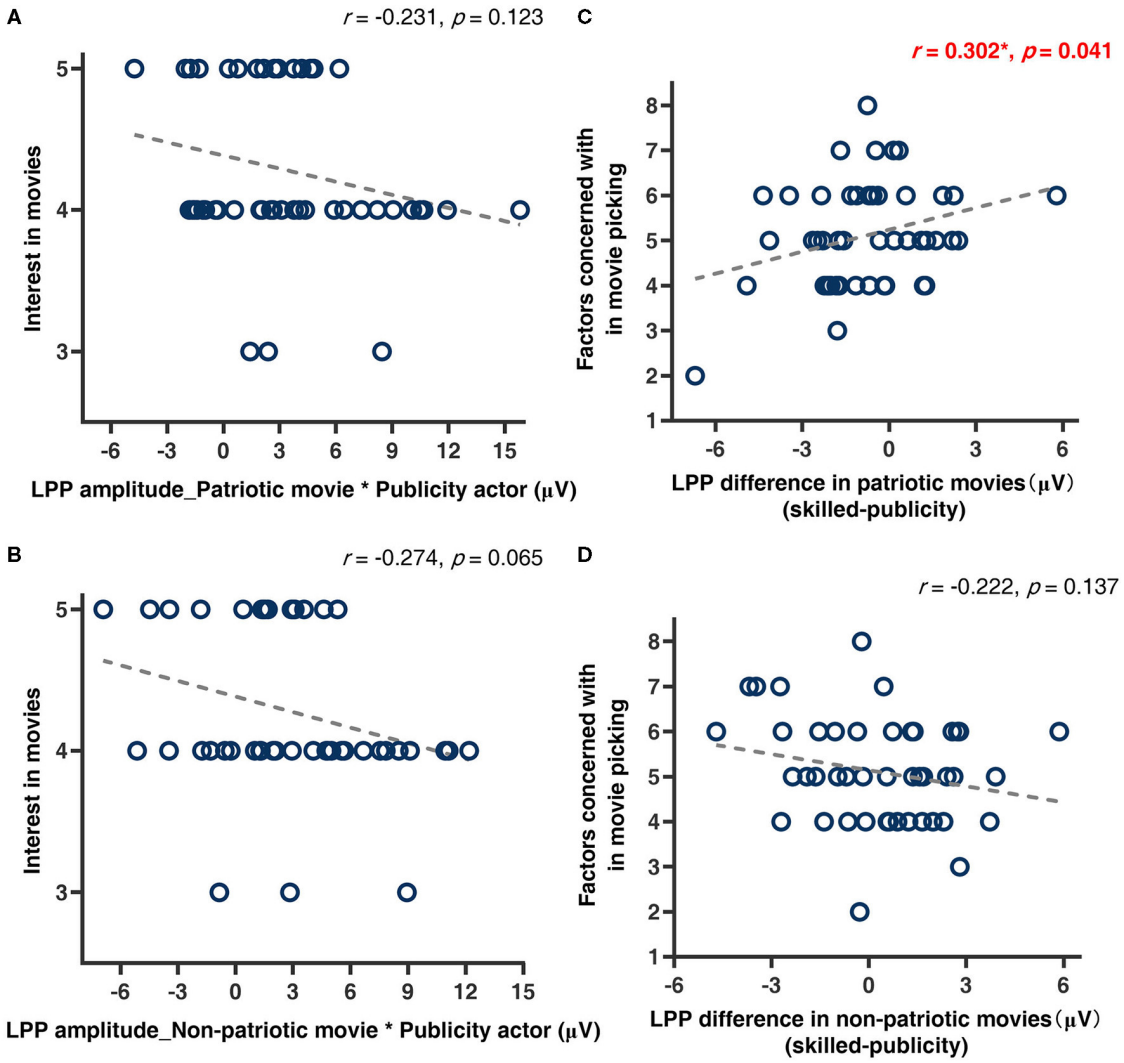
Scatter plots and Pearson's correlation coefficient analysis results. **(A,B)** Correlations between average LPP amplitude and the degree of interest in movies under publicity actor conditions. **(C,D)** Correlations between LPP amplitude difference (skilled–publicity) in different movie types and the number of factors each participant concerned with in movie picking.

Correlation analyses were also performed on LPP to investigate relationships between watching habits and conflicted emotional responses. When it came to the publicity actor condition, no significant correlation was found between LPP amplitude and the interest degrees in patriotic (*p* = 0.123, Figure 5A) and non-patriotic movies (*p* = 0.065, Figure 5B). Another interesting finding was that the number of attributes each participant identified (e.g., actor, script, director, movie genres, and so forth) in selecting movies was positively correlated with the LPP amplitude difference (skilled–publicity) in patriotic movies (*r* = 0.30, *p* = 0.041, Figure 5C). This meant that the more factors the participant took into account in selecting a movie, the larger the LPP amplitude difference she had. In contrast, no significant correlation was found in non-patriotic movies (*p* = 0.137, Figure 5D).

### Theta ERS Results

**Figure 6:**
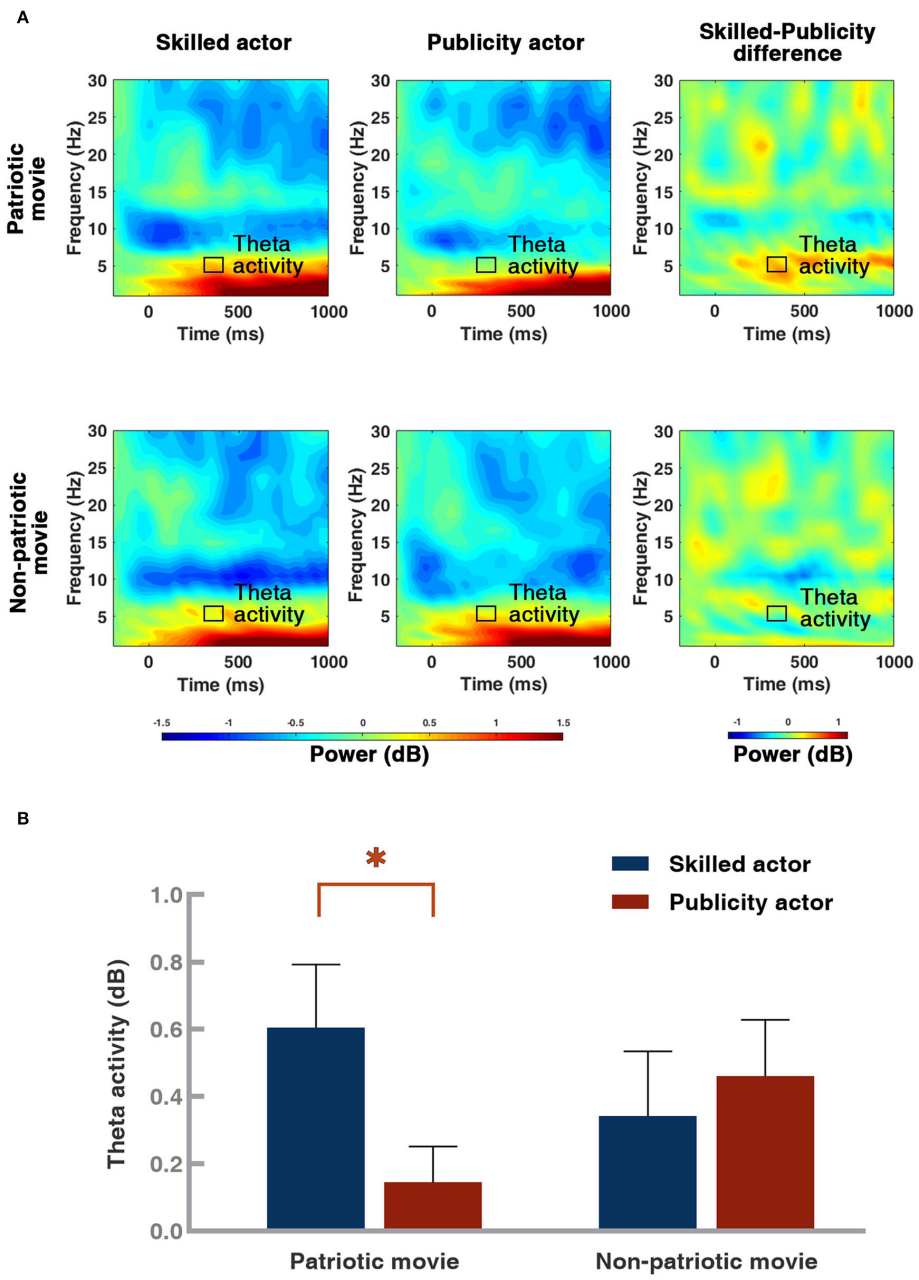
Illustration of neural oscillation at Fz and theta ERS results. **(A)** Illustration of grand-averaged neural oscillation at Fz under four conditions and skilled/publicity difference. **(B)** Averaged magnitude of theta activity from Fz (frequency range = 4.1–5.8 Hz, time range = 330–380 ms) under four conditions. Theta band (4–7 Hz) activity from 330 to 380 ms, with a frequency range of 4.08–5.80 Hz, was selected (the black box area). In the graphs, the shade of the color represents the strength of the power. The darker the color was, the stronger was the power. Error bar: standard error, *p < 0.05.

In accordance with previous studies of theta band oscillation (Aftanas and Golocheikine, 2001; Kubota et al., 2001), the mean magnitude of FM-theta-band activity (Fz) (Yamamoto and Matsuoka, 1990; Mitchell et al., 2008) with the 330–380 ms time-window (Kolev et al., 1997; Yordanova and Kolev, 1998; Wang and Ding, 2011) was chosen for analysis (see in Figure 6A). A 2 (movie type: patriotic vs. non-patriotic) × 2 (actor type: skilled vs. publicity) ANOVA was conducted. The ANOVA result (Figure 6B) showed insignificant main effects of movie type [*F*_(1, 45)_ = 0.042, *p* = 0.839] and actor type [*F*_(1, 45)_ = 1.886, *p* = 0.176]. There was a significant interaction between the movie type and the actor type [*F*_(1, 45)_ = 5.543, *p* = 0.023]. The further simple effect test indicated an enhanced theta-band activity for skilled actors (0.61 ± 0.19 dB) compared to publicity actors (0.14 ± 0.17 dB) in patriotic movies [*F*_(1, 45)_ = 7.136, *p* = 0.010]. On the contrary, there was no significant difference between actor types in non-patriotic movies [*F*_(1, 45)_ 0.443 1, *p* = 0.509]. Results of behavior and brain activities are summarized in Table 1.

**Table 1:** The behavioral and brain results.

**Behavioral results (movie-watching willingness)**		ANOVA	ME-Movie type		N/A		
			ME-Actor type		SA > PA		
			Interaction effect	PM			SA > PA
				NM			SA > PA
		Pearson's correlation	Willingness growth caused by skilled actors	and	Familiarity with patriotic movies		Positive
Brain results	P3 amplitude	ANOVA	ME-Movie type	PM > NM			
			ME-Actor type	N/A			
			Interaction effect	PM			SA < PA
				NM			N/A
		Pearson's correlation	N/A				
	LPP amplitude	ANOVA	ME-Movie type	N/A			
			ME-Actor type	N/A			
			Interaction effect	PM			SA < PA
				NM			N/A
		Pearson's correlation	Interest degree in movies	and	LPP amplitude (PA)	PM	N/A
						NM	N/A
			Factors participants concerned with in movie-picking	and	LPP difference (SA-PA)	PM	Positive
						NM	N/A
	Theta ERS	ANOVA	ME-Movie type	N/A			
			ME-Actor type	N/A			
			Interaction effect	PM			SA > PA
				NM			N/A
		Pearson's correlation	N/A				

*N/A, this effect is insignificant. ME-Movie type, Main effect of movie type; ME-Actor type, Main effect of actor type; PM, Patriotic movie; NM, Non-patriotic movie; SA, Skilled actor; PA, Publicity actor*.

## Discussion

This exploratory study set out to examine audience preferences on skilled or publicity actors for patriotic movies by using EEG. Plenty of previous studies have confirmed that the presence of actors in movies influence the choices of an audience one way or another (Faulkner and Anderson, 1987; Levy, 1990; Albert, 1998; Rein et al., 2006; Hadida, 2010; Liu et al., 2014; Joshi, 2015; Peng et al., 2019). Our behavioral data indicated that the willingness of an audience to watch a movie was higher for skilled actors than for publicity actors, especially for patriotic movies. This is consistent with the study by Yang on patriotic movies, arguing that patriotic movies need strong skilled actors to lift the artistic quality and, in turn, appeal to a large audience (Yang, 2017). Using skilled actors to attract audiences for better market performance was regarded as more important in less familiar genres than in popular genre movies, such as comedies and dramas (Desai and Basuroy, 2005). Audiences are less familiar with patriotic movies as a genre than with movies in which patriotism is not an issue, and this leads to a greater preference for skilled stars in this study. Further, the study further discovered that the more familiar the participant was with patriotic movies, the stronger preference she had for skilled actors than for publicity actors. This was also in line with previous studies, which suggested that more experienced moviegoers are more likely to be attracted by skilled stars with their outstanding acting abilities (Hofmann, 2019) and reliable film quality marker function (Franck, 2001). This led us to conclude that this type of audience puts a stronger emphasis on the artistic quality of the movies.

Following the behavioral results, our EEG experiment drew a similar picture. First, the ERP results showed that, as hypothesized, audiences had smaller P3 and LPP amplitude for skilled actors than for publicity actors in the patriotic movie condition. It has been argued that P3 is related to attentional salience (Palomba et al., 1997; Cuthbert et al., 2000; Di Russo et al., 2006; Hajcak et al., 2010), while LPP reflects the degree of emotional arousal (Cuthbert et al., 2000; Schupp et al., 2003, 2007; Hajcak et al., 2006). This result supported our hypothesis that the motivational salience and emotional arousal toward skilled actors are lower in the patriotic movie condition. In other words, people were more likely to be emotionally engaged with a patriotic movie if it stars a skilled actor, and this enhances their willingness to watch this type of movie. This was consistent with our behavioral results. Further, the correlation between the number of movie attributes that participants focused on when selecting a movie and the different LPP amplitude caused by the two types of actors also confirmed this, suggesting that in patriotic movies, the performance of skilled actors can reduce the concerns of an audience about the script, props, and film type to a certain extent and make it easier to attract an audience. This further emphasizes the importance for a patriotic movie to hire skilled actors in order to have a positive influence on the audience.

Furthermore, as we have proposed, a larger theta oscillation was observed for the skilled actors than for the publicity actors in the patriotic movie condition. Since a larger theta ERS is induced by emotionally positive stimuli (Aftanas et al., 1998; Aftanas and Golocheikine, 2001; Sammler et al., 2007), our results confirmed that participants have more favorable emotional reactions to skilled actors when they watch a patriotic movie. This replicated the behavioral and ERP results of this study.

Taken together, both behavioral evidence and neural evidence from this study prove that the willingness of an audience to watch a patriotic movie is deeply affected by actor types. Specifically, skilled actors engage the audience emotionally, more so than publicity actors, and significantly increase the likelihood of the audience choosing patriotic movies. In particular, more experienced audience members showed a stronger preference for skilled actors. Furthermore, apart from acting as quality markers, skilled actors in patriotic movies also help distract people from other attributes of the movies, making them a more valuable asset for this type of production (Hofmann, 2019).

The current study may have a couple of theoretical implications. First, this study reveals the psychological and neural mechanism of how the willingness of an audience to watch patriotic movies is influenced by actor types. As argued before, screen images of publicity stars associated with the light comedies they appear in, and the daily gossip they generate, may distract people from the storyline of patriotic movies (Hofmann and Opitz, 2019) and induce a cognitive conflict for the audience. In addition, this study provides neurological evidence that patriotic movies should cast skilled actors to better engage audiences emotionally. Second, this is the first attempt in film study to explore the relationship between the willingness of audiences to watch a movie and their preferences for actor types by using neuroscience technology, revealing a close correlation between behaviors of audiences and their brain activities. These findings advance film study with careful scientific measurements and a possible new direction.

A bedrock issue in film studies concerns the use of empirical studies to guide production (Wallace et al., 1993). This study also has a couple of practical implications. First, the study provides a new perspective for the delivery of patriotism through cinemas by focusing on the mechanism of elevating the willingness of audiences to watch a patriotic movie. It suggests that the astute choice of suitable actors can enhance attention and positive emotional reaction of audiences to the patriotic movie, leading to large dissemination of the values and ideas dramatized. Second, this study, supported by clear neuroscientific evidence, provides the film industry with an effective tool to select actors for better audience engagement. This can be a useful alternative to practical industrial experience and findings from previous studies.

However, there are also some issues arising from this study that should be addressed in future research. First, we only selected female participants in this study. This is because previous studies have found that, in general, women have less interest in patriotic movies that often involve war, historical events, and political figures than men (Hsu, 2006; Kord and Krimmer, 2011), which means there is a greater urgency in studying the reactions of female audiences. To compensate, we used only male actors as stimuli in our experiment. As Addis and Holbrook (2010) have pointed out, audiences tend to orient their preferences toward opposite-gender stars. Future studies could include male participants and female actors in the material to explore possible differences in behavioral and neural reactions. Second, the experiments in this study were conducted with individual participants. However, movie watching can be a complex group behavior, which may not be fully comprehensible purely by studying the reactions of individual brains. For future studies, it is worth using hyper-scanning technology to further explore group dynamics among different audiences.

## Data Availability Statement

The raw data supporting the conclusions of this article will be made available by the authors, without undue reservation.

## Ethics Statement

The studies involving human participants were reviewed and approved by Laboratory of Applied Brain and Cognitive Sciences, Shanghai International Studies University. The patients/participants provided their written informed consent to participate in this study.

## Author Contributions

LZ conceived the experiment and developed the project. YW conducted the experiment and analyzed the results. LZ and YW wrote the first draft. All authors reviewed the manuscript and contributed to the submitted version.

## Conflict of Interest

The authors declare that the research was conducted in the absence of any commercial or financial relationships that could be construed as a potential conflict of interest.
